# Genetic and Chromosomal Aberrations and Their Clinical Significance in Renal Neoplasms

**DOI:** 10.1155/2015/476508

**Published:** 2015-09-13

**Authors:** Ning Yi Yap, Retnagowri Rajandram, Keng Lim Ng, Jayalakshmi Pailoor, Ahmad Fadzli, Glenda Carolyn Gobe

**Affiliations:** ^1^Department of Surgery, Faculty of Medicine, University of Malaya, Jalan Universiti, Kuala Lumpur, Malaysia; ^2^Centre for Kidney Disease Research, University of Queensland, Translational Research Institute, 37 Kent Street, Brisbane QLD 4102, Australia; ^3^University of Malaya Cancer Research Institute, University of Malaya, Jalan Universiti, Kuala Lumpur, Malaysia; ^4^Department of Pathology, Faculty of Medicine, University of Malaya, Jalan Universiti, Kuala Lumpur, Malaysia

## Abstract

The most common form of malignant renal neoplasms is renal cell carcinoma (RCC), which is classified into several different subtypes based on the histomorphological features. However, overlaps in these characteristics may present difficulties in the accurate diagnosis of these subtypes, which have different clinical outcomes. Genomic and molecular studies have revealed unique genetic aberrations in each subtype. Knowledge of these genetic changes in hereditary and sporadic renal neoplasms has given an insight into the various proteins and signalling pathways involved in tumour formation and progression. In this review, the genetic aberrations characteristic to each renal neoplasm subtype are evaluated along with the associated protein products and affected pathways. The potential applications of these genetic aberrations and proteins as diagnostic tools, prognostic markers, or therapeutic targets are also assessed.

## 1. Introduction

The incidence of kidney cancers has been increasing steadily in developed countries over the past decade and new reports show similar trends in developing countries [1–3]. Renal cell carcinoma (RCCs) form the majority of malignant kidney neoplasms and are grouped into a few different subtypes based on their histomorphological features. The most common subtype is clear cell RCC (ccRCC), followed by papillary (pRCC), chromophobe (chRCC), and collecting duct RCC (CDRCC). Renal oncocytoma (RO) is a benign renal neoplasm with histological similarities to chRCC [4]. Accurate identification of these subtypes is important for disease management as each subtype has a different biological and clinical behaviour. Cancer is often described as a genetic disease, caused by genetic alterations which regulate cell growth [5–7]. Hence, distinctive genetic aberrations in each RCC subtype affect the clinical course and prognosis of the tumour. The majority of RCCs are sporadic while only approximately <3% are hereditary [8, 9]. Although less frequently encountered, hereditary RCCs play a significant role in the understanding of genetic changes and pathways affecting tumour progression (Table 2).

Genetic alterations such as duplication, deletion, translocation, hypermethylation, or mutations result in the activation or inactivation of genes and the over- or underexpression of the corresponding proteins in RCCs. Genetics analysis helps in the identification of tumours in situations where histology and immunohistochemistry (IHC) profiles do not provide a clear distinction between the subtypes. Conventional methods for analysis include chromosomal comparative genomic hybridization (CGH), fluorescent in situ hybridization (FISH), G banding, and polymerase chain reaction (PCR) based loss of heterozygosity (LOH) analysis. Newer technologies such as array CGH (aCGH), single nucleotide polymorphisms (SNP) arrays, and next generation sequencing (NGS) have allowed for high throughput analyses of known aberrations as well as identification of novel genetic alterations.

In this review, the various genetic aberrations associated with familial and sporadic clear cell, papillary, chromophobe, collecting duct RCCs, and oncocytoma will be explored, along with the prognostic, diagnostic, and therapeutic implications of these genes.

## 2. Clear Cell Renal Cell Carcinoma (ccRCC)

Clear cell RCC is the most frequent subtype of renal cancer, accounting for 80–90% of all RCCs [10]. Originating from renal proximal tubules, ccRCC cells have abundant clear cytoplasm, a result of high glycogen and lipid content. Eosinophilic or granular cells may be present, due to a higher number of mitochondria [11]. Clear cell RCC has a metastatic rate of 15.3–21.5% at presentation and a 5-year cancer specific survival of 71–81.3% [12–14].

### 2.1. Von Hippel-Lindau (VHL) Gene

The most common and characteristic genetic changes in ccRCCs are aberrations such as LOH, hypermethylation, or mutation in 3p chromosome region, which are found in up to 91% of the tumours [15–17]. The* VHL* gene, a tumour suppressor gene (TSG) located at 3p25 loci, is one of the earlier genes associated with ccRCC. This gene was first discovered in patients with VHL disease, an autosomal dominant hereditary syndrome that predisposes affected individuals to cancers such as pheochromocytomas, hemangioblastomas, and ccRCC [18]. Up to 75% of patients with VHL syndrome develop ccRCC, a leading cause of death in these patients [19, 20]. However, only approximately 1.6% of all ccRCC cases are associated with hereditary VHL syndrome [21]. Interest in* VHL* grew when the majority of sporadic ccRCC cases were also found to contain* VHL* aberrations.

The VHL protein (pVHL) functions to ubiquitinate proteins and mark them for degradation, with hypoxia inducible factor *α* (HIF*α*) being a well-researched target involved in tumourigenesis [22–24]. HIF*α* is a transcription factor controlling angiogenesis, glucose uptake, cell proliferation, and apoptosis through downstream targets like vascular endothelial growth factor (VEGF), platelet-derived growth factor (PDGF), transforming growth factor alpha (TGF*α*), and C-X-C chemokine receptor type 4 (CXCR4) [25–27]. Under normal oxygen conditions, HIF*α* is degraded by pVHL but when oxygen is low, HIF*α* is allowed to accumulate, inducing transcription of genes that improve cell proliferation, oxygen delivery, and angiogenesis [22–24]. Inactivation of* VHL*, which occurs through mutation, deletion, or methylation, causes the accumulation of HIF*α* under normal oxygen conditions and encourages tumour growth. Individuals with VHL disease have one wild type* VHL* allele and one inactivated* VHL* allele [28]. According to the Knudson two-hit model, biallelic VHL inactivation is achieved when the remaining copy is inactivated by spontaneous mutation [28, 29]. Sporadic renal tumours require spontaneous mutation of two wild type* VHL* alleles [30].

*VHL* genetic changes are believed to be exclusively associated with ccRCC but van Houwelingen and colleagues have detected* VHL* mutations in 15% of non-ccRCCs from a cohort of sporadic RCC patients in Netherlands. They noted that the percentage of patients with* VHL* mutation was significantly higher for ccRCC compared to RCCs of other histological types [31]. Other studies however found no* VHL* mutation in other subtypes [32–34].

Inactivation of* VHL* may play an important role in the pathogenesis of ccRCC, but the association of* VHL* status with clinicopathological parameters and disease progression is still unclear and contradictory. Some studies showed that the presence or absence of* VHL* alterations does not affect tumour stage, grade, or prognosis [35–37]. It has also been shown that there is no correlation between* VHL* mutation or methylation status with angiogenesis and proliferation of ccRCC [35, 38]. Brauch et al. and Schraml et al. reported an association of tumour* VHL* alterations with advanced stage and adverse prognosis [35, 39]. In contrast, several other studies revealed favourable prognosis for tumours with* VHL* inactivation or alteration [40–44]. These contradictory prognostic findings could be a result of complex transcriptional or posttranscriptional responses in addition to the various genetic aberrations which contribute to the heterogeneous characteristics of RCCs [35]. It is postulated that the* VHL* independent activation of other signal transduction pathways such as the mammalian target of rapamycin (mTOR), nuclear factor kappa-light-chain-enhancer of activated B cells (NF-*κ*B), signal transducer and activator of transcription 3 (STAT-3), or epidermal growth factor receptor (EGFR) pathways could be responsible for tumour progression and tumours activated by several different mechanisms could be more aggressive [42]. Moreover, ccRCC tumours caused by VHL syndrome are of lower grade, less likely to metastasize, and have better 10-year survival compared to sporadic* VHL* RCCs, which may harbour other predisposing mutations [21].

Nevertheless, the discovery of the* VHL* gene in familial and sporadic ccRCC has revolutionised treatment for advanced RCC. Targeted therapy aiming at suppressing angiogenesis through VEGF or PDGF mediated pathways has replaced immunotherapy such as interferon alpha (IFN*α*) and interleukin-2 (IL-2) as treatment for metastatic RCC. The current FDA approved targeted therapy drugs for RCC which are the tyrosine kinase inhibitors (sunitinib, sorafenib, pazopanib, and axitinib), monoclonal antibody to VEGF (bevacizumab), and the mTOR inhibitors (temsirolimus and everolimus) [45]. Targeted therapy has improved treatment outcome as the overall and cancer specific survival of metastatic RCC patients has improved in the targeted therapy era compared to the immunotherapy era [46, 47].

### 2.2. Chromosome 3 Translocations

A number of studies have reported hereditary ccRCC associated with translocation of chromosome 3, thus supporting the role of chromosome 3 in the pathogenesis of ccRCC. Cohen et al. (1979) first discovered a reciprocal translocation at (3;8)(p14.2;q24) in a family with hereditary ccRCC [48]. A possible gene of interest,* FHIT*, has been identified in that chromosome 3 region, which will be discussed in the next section. Since then, other constitutional translocations in chromosome 3 regions have been reported. These translocations such as t(1;3)(q32;q13), t(2;3)(q33;q21), t(2;3)(q35;q21), t(3;6)(q12;q15), and t(3;8)(p13;q24) were found in familial ccRCC [49–54]. In some familial chromosome 3 translocations identified, t(3;4)(p13;p16), t(3;4)(q21;q13), t(3;6)(p13;q25), and t(3;15)(p11;q21), only one family member developed ccRCC [55–57]. The involvement of several TSG genes has been suggested, for example, t(1;3)(q32;q13) (*NORE1* and* LSAMP*), t(2;3)(q33;q21) (*DIRC1*), t(2;3)(q35;q21) (*DIRC2* and* DIRC3*), and t(3;8)(p14.2;q24) (*FHIT* and* TRC8*). However, these translocations result from different breakpoints and some translocations had no identifiable breakpoint associated genes [52, 53, 58]. Hence, a three-step model has been proposed for familial chromosome 3 translocation ccRCC. The first step is inherited translocation of chromosome 3, followed by the loss of the 3p segment, and finally somatic mutation of the remaining 3p allele, which may contain the VHL gene or other TSGs located at 3p [54, 59].

### 2.3. Fragile Histidine Triad (*FHIT*)

Although* VHL* plays a significant role in our understanding of ccRCC, it is not the only genetic aberration in chromosome 3p region. In ccRCC with 3p deletion with and without* VHL* alterations, inactivation of TSGs at 3p12-p21 appears to play a role in tumourigenesis [33, 60]. Numerous studies have been carried out to identify potential TSGs along this region. One such gene is* FHIT* located in the 3p14.2 region. Chromosomal translocation at t(3;8)(p14.2;q24) was first described in hereditary RCC and a common region of loss in chromosome 3 was found in sporadic ccRCC [61–64].* FHIT* gene has been identified at this locus in several other malignancies including lung, breast, cervical, gastric, and bladder cancers [65–69]. The* FHIT* gene encompasses the chromosomal fragile site FRA3B, a frequently observed breakpoint in many cancers including RCC [70, 71]. As a result of inactivation, FHIT protein expression is low or absent in most ccRCC tumours [72–74].

The specific function of the FHIT protein is still unclear, but studies have demonstrated the role of the* FHIT* gene in tumour suppression. For example, there was an increase in formation of spontaneous tumours and susceptibility to carcinogen-induced tumours in* FHIT* knockout mice compared to mice with functional* FHIT* [75, 76]. There is a significant correlation between low or absence of FHIT protein expression and low grade and early stage ccRCC tumours indicating that LOH of FHIT may play a role in early tumour development [73, 74, 77, 78]. Interestingly, Ramp et al. reported that higher FHIT protein expression is linked to poorer patient survival and is an independent prognostic marker in ccRCCs [73]. Although FHIT is lower in ccRCC compared to normal kidney, higher FHIT levels in higher grade and stage tumours do not support the tumour suppressor function of FHIT [72–74]. Ramp et al. suggested that reversible epigenetic inactivation such as gene hypermethylation or posttranslational events may reactivate FHIT as the tumour progresses [73, 77]. However, there has yet to be conclusive evidence for these mechanisms.

### 2.4. Ras Association Domain Family 1A (*RASSF1A*)

Another TSG associated with ccRCC in 3p chromosome region is* RASSF1A* gene located at 3p21.3. RASS1A protein regulates microtubule formation, cell cycle control, and apoptosis [79]. Reintroduction of RASSF1A in lung and breast cancer cell lines inhibited cell cycle progression and proliferation [80].* RASS1A* is inactivated by hypermethylation in the promoter region and this is frequently seen in ovarian, breast, and lung tumours, including ccRCC [81–83]. Peters et al. found significantly increased methylation in ccRCC tumours compared to normal tissues but also detected a subgroup of methylated sequences in the normal tissue [84]. The normal tissue was obtained from histologically benign region of the tumour bearing kidney, suggesting that hypermethylation of* RASSF1A* is involved in early tumour formation of RCC [84]. In ccRCC patients, hypermethylation of the* RASSF1A* promoter was significantly associated with advanced stage, higher grade, and unfavorable patient survival [85, 86]. Tezval et al. reported that most ccRCC tumours have low RASS1A protein expression but a subset of tumours with increased expression is associated with higher stage and grade [87]. This is somewhat contradictory to the tumour suppressor function of the protein. More studies on protein expression of RASSF1A in ccRCC tumour tissue are needed to understand its effect on patient prognosis. Although less frequently reported,* RASSF1A* inactivation is also found in approximately 44% of papillary RCC [88].

### 2.5. Chromatin Modification Genes

Recently, NGS or exome sequencing studies have discovered several novel genes involved in chromatin modification which are mutated in ccRCC [89–91]. The newly identified genes are polybromo-1 (*PBRM1*), AT-rich interactive domain-containing protein 1A (*ARID1A*), BRCA1 associated protein-1 (*BAP1*), SET domain-containing 2 (*SETD2*), and lysine- (K-) specific demethylase 5C (*KDM5C*) [89–93].* PBRM1* mutations are found in up to 41% of ccRCC, making it the second most mutated gene after* VHL* [90].* PBRM1*,* BAP1*, and* SETD2* are all located near the 3p21 region and, similar to* VHL*, are proposed to be inactivated through the Knudson two-hit model [94]. As loss or deletion of 3p chromosome region is common in ccRCC, inactivation of these genes is achieved by further mutations in the remaining allele [90].

The discovery of these frequently mutated genes along with advances in NGS technology has led to interest in intratumour heterogeneity analysis in ccRCC. This provides an insight into the various mutations that may take part in tumour initiation or progression and possibly aid in fine tuning targeted therapy in the future. Multiple regions from a single tumour region were subjected to sequencing and mutations were mapped onto a phylogenic tree to illustrate the evolution of mutational events. Early or ubiquitous mutations are located at the trunk while subsequent or intratumour subclonal mutations are located at the branches. A ubiquitous mutation is found in all regions of a tumour analysed, whereas subclonal mutations are only found in a subpopulation of tumour cells [94, 95].

*VHL* was mapped as a ubiquitous driver mutation in 64–100% of ccRCC tumours analysed by Gerlinger et al. and Sankin et al. [95, 96].* BAP1*,* PBRM1*,* SETD2*, and* KDM5C* mutations were found in different subclones in the same tumour, indicating that these were later mutations [95, 96]. However,* PBRM1* was also a ubiquitous mutation in 20–30% of ccRCC tumours analysed [95, 96]. Sankin et al. reported an increased mutation frequency in intratumour multiregion analysis, compared to earlier large scale sequencing studies [96]. This was especially observed in nonubiquitous mutations, such as* SETD* and* KDM5C* [96]. They suggested that the actual mutation rate may be higher than reported in earlier studies, but this will have to be validated in a larger sample sized multiregion sequencing analysis. Although most studies have focused on mutations of these genes in sporadic ccRCC, germline* PBRM1* and* BAP1* mutations have been detected in familial ccRCC [97, 98]. Germline* BAP1* mutation also predisposes affected individuals to uveal melanoma, malignant pleural mesothelioma, and cutaneous melanoma [99].

These chromatin modification genes function as TSGs and have been implicated in other cancers, such as* ARID1A* and* SETD2* in breast cancer and* KDM5C* in prostate cancer [100–102]. Alterations of the chromatin modification proteins could lead to disruptions of transcriptional regulation and tumour formation [92]. For example, silencing of* PBRM1* in ccRCC cell lines increased proliferation and migration, supporting its role as a TSG [90]. In a RCC cell line with* BAP1* mutation, 769-P, cell proliferation was inhibited by the introduction of wild type* BAP1* [91].

The roles of these chromatin modification genes and their proteins products are not fully understood yet, but various studies have shown that the mutational status of these genes may possess prognostic influence on ccRCC. Low PBRM1 expression was reported to correlate with advanced stage, higher Fuhrman grade, and worse disease specific survival [103, 104]. In contrast, Hakimi et al. and Sato et al. found no relationship between disease-free or disease specific survival and* PBRM1* mutational status [105, 106]. BAP1 loss was associated with metastasis, advanced stage and Fuhrman grade, sarcomatoid differentiation, and worse overall and disease specific survival [91, 105, 107, 108]. Compared to tumours exclusively mutated for* PBRM1*, tumours with* BAP1* only mutation conferred adverse clinicopathological features and prognosis [109, 110]. Based on these findings,* BAP1 *mutational status appears to be a strong prognostic indicator for ccRCC. Low ARID1A mRNA and BAF250a (protein product of* ARID1A*) levels also correlated with higher stage, grade, and worse prognosis while* SETD2* mutation was associated with worse disease specific survival [93, 105, 111].

### 2.6. Other Genetic Aberrations

Other genetic aberrations of interest, such as changes at chromosome regions 5q, 8p, 9p, and 14, may affect the prognosis of ccRCC. Copy number gains at 5q conferred a favourable prognosis whereas a loss had an adverse effect [112–114]. LOH in 8p, 9p, and 14q has been associated with higher grade, stage, unfavourable prognosis, and tumour recurrence [115–121]. Potential candidate genes include* CDK2NA* (cyclin-dependent kinase inhibitor 2A) at 9p21 and* HIF1A* at 14q23.2 [118, 119, 121].* CDK2NA* encodes the p16 tumour suppressor protein which plays an important role in cell cycle regulation by preventing cells progressing from G1 phase to S phase [122].

Recent sequencing, transcriptome, and integrated data analyses have revealed frequently mutated signalling pathways in ccRCC such as the phosphoinositide 3-kinase PI3K-AKT-mTOR and p53 pathways [106, 123]. The PI3K-AKT-mTOR pathway, which regulates angiogenesis, cell cycle progression, and proliferation, is a target for mTOR inhibitors temsirolimus and everolimus. Together, genes involved in the pathway, consisting of* MTOR*,* PTEN*,* PIK3CA*,* AKT2*, and others, have alterations in 26–28% of ccRCC tumours [106, 123]. These findings provide concrete evidence on the genetic changes directly associated with the PI3K-AKT-mTOR pathway in ccRCC. Targeted therapy with mTOR inhibitors in RCC was based on the understanding that elevated HIF could induce the PI3K/AKT pathway and promote angiogenesis [124]. Alterations of the genes found in the p53 signalling pathway, which includes* TP53* and* CDK2NA*, could be detected in 40% of tumours [106]. The p53 pathway plays a role in apoptosis, inhibition of angiogenesis, and genomic stability. Knowledge in deregulated signalling pathways would help in providing patients with personalised targeted therapy and improving treatment outcome.

*Differential Diagnosis*

*Clear Cell Tubulopapillary RCC*. Clear cell tubulopapillary RCC is a recently identified RCC subtype comprising of cells with clear cytoplasm and papillary architecture. It is often reported in patients with end stage kidney disease but has been found in normal functioning kidneys and is typically low grade [125]. Although clear cell tubulopapillary RCC mimics ccRCC and pRCC histologically, it has a different genetic profile from both subtypes [126]. Clear cell tubulopapillary RCCs lack deletions of 3p typical of ccRCC or gains of chromosome 7 and loss of chromosome Y characteristic of pRCC [126–128]. In addition, clear cell tubulopapillary RCC lacks* VHL* mutation commonly found in the ccRCC subtype [129].

## 3. Papillary Renal Cell Carcinoma (pRCC)

Papillary RCC is the second most common subtype, making up approximately 10% of all RCCs. Papillary RCC is characterised by tumour cells arranged in tubulopapillary architecture. Delahunt and Eble have proposed the subclassification of pRCCs into two histological groups, type 1 and type 2 [130]. Type 1 tumours are characterised by small cells with scant cytoplasm and small nuclei while type 2 tumours have large cells with plentiful eosinophilic cytoplasm and large spherical nuclei. Type 2 tumours are generally more aggressive, with more advanced grade and stage than type 1 tumours [130, 131]. Compared to ccRCC, pRCCs have a better prognosis with a metastatic rate of 3.4–14.9% at presentation and 5-year cancer specific survival of 79.4–91% [12–14].

### 3.1. Chromosomes 7 and 17

Papillary RCCs frequently display gains of chromosomes 7 and 17 [132–134]. Trisomies 7 and 17 discovered in small papillary renal cell neoplasia indicate that these genetic alterations may be involved in initial tumour development [135]. Based on past studies, polysomy 7 is not a reliable predictor of survival, stage, grade, or proliferation rate of papillary renal tumours [132, 133, 136, 137]. Polysomy 7 in ccRCC however was correlated with higher stage and grade [133]. The prognostic value of polysomy 17 in pRCC is contradictory. Balint et al. found no significant association between chromosome 17 gains and tumour grade or size [132]. In contrast, Gunawan et al. and Klatte et al. significantly correlated chromosome 17 polysomy with lower stage, less lymph node, and distant metastases, as well as a favourable survival [137, 138].

### 3.2. MET Protooncogene

At present, only one gene on chromosome 7 has been positively identified and linked to pRCC. Hereditary pRCC (HPRC) associated with type 1 tumours is caused by the mutation of the* MET* protooncogene at 7q31. An activating missense mutation of the* MET* gene and duplication of chromosome 7 along with the mutated* MET* gene were postulated to increase the oncogenic effect of* MET* [139, 140].* MET* mutation associated hereditary pRCC and sporadic pRCC are typically low grade, bilateral tumours with multiple lesions [8, 9, 141]. The protein product of the* MET* gene is c-met, a hepatocyte growth factor receptor (HGFR). Binding of the hepatocyte growth factor (HGF) or c-met to the MET receptor induces several biological responses which promote oncogenesis, including cell motility, cell differentiation, proliferation, angiogenesis, and invasion [142]. The role of c-met in pRCC has not been clearly elucidated but in hereditary pRCC it is suggested that germline mutations of the* MET* gene promote proliferation, tubulogenesis, and tumour initiation [143].

*MET* mutation is estimated to be present in 5–21.6% of sporadic pRCC [8, 108, 139, 141]. A recent study by Albiges et al. reported copy number gains of MET in 46% of type II pRCC and in 81% of type I pRCC [108]. The c-met protein is strongly expressed in 80–90% of pRCC, indicating a role of* MET* copy number gains in protein activation [108, 143, 144]. Interestingly, there is no significant difference between c-met expressions in type 1 and type 2 pRCC although tumours with MET mutations favour the type 1 histology [141, 143, 144]. Sweeney et al. found a significant correlation between c-met expression and higher tumour stage and a trend towards a favourable overall survival rate for patients with c-met negative tumours [143]. To date, no other studies have shown prognostic association of c-met protein or* MET* gene in pRCC. In contrast, c-met expression is lower in ccRCC but studies have shown significant correlation of positive c-met immunoreactivity with metastasis and higher grade and stage [144, 145]. A recent study by Gibney et al. reported a correlation of higher c-met expression with higher grade and stage and worse disease specific survival in RCCs [146]. This may not be specific to pRCCs as they analysed all RCC subtypes, including a majority of ccRCC [146]. Regardless of the prognostic significance, the c-met signalling pathway appears to be an attractive target for pRCC. Phase II clinical trials of c-met pathway inhibitors, volitinib and foretinib, are currently ongoing for advanced pRCC [147, 148].

### 3.3. Fumarate Hydratase (*FH*)

Besides* MET* mutation associated HPRC, another form of hereditary pRCC is found in the autosomal dominant syndrome, hereditary leiomyomatosis, and renal cell carcinoma (HLRCC). Affected individuals are predisposed to develop cutaneous leiomyomas, uterine fibroids, and type 2 pRCC [149]. Renal tumours in HLRCC patients are characteristically solitary and unilateral with a propensity for nodal or distant metastasis [150, 151]. In rare cases, renal tumours are of collecting duct RCC histology [149, 152]. Germline mutation of the* FH* gene has been identified and mapped to chromosome 1q42-43 [152, 153]. Fumarate hydratase (FH) protein is an enzyme responsible for converting fumarate to malate in the Krebs cycle. As a consequence of missense, frameshift, insertion/deletion, nonsense, or complete deletions of the* FH* gene, enzymatic activity of FH is significantly decreased [154]. Loss of FH activity causes the accumulation of fumarate, which can act as a competitive inhibitor of HIF prolyl hydroxylase (HPH) [155]. HPH degrades hypoxia inducible factors (HIFs) in normoxia conditions; hence elevated levels of fumarate result in stabilization and accumulation of HIFs [155]. Elevated HIFs promote angiogenesis and tumour progression. Compared to* VHL* and* MET*, mutation of* FH* in sporadic RCC is very rare [156]. For example, according to the COSMIC (Catalogue of Somatic Mutations in Cancer) database,* FH* mutations were found in 3 out of 1383 renal tumours analysed [157].

### 3.4. Other Genetic Aberrations

Deletions are often found in chromosomes X and Y of pRCC tumours. The effects of these chromosomal losses in pRCC are not well documented in literature and no TSG has been identified yet. Only one study, by Jiang et al., has significantly linked losses of chromosome Xp with shorter patient survival [158]. Besides gains of chromosomes 7 and 17, gains of chromosomes 12, 16, and 20 and loss of chromosome Y have also been detected in renal papillary adenomas, suggesting that these chromosomal alterations may be involved in early neoplastic changes in pRCC [159, 160]. Multiregion sequencing analysis confirmed that gains in chromosomes 7, 12, 16, and 17 are ubiquitous early events in pRCC tumourigenesis [161].* BAP1*,* SETD2*,* ARID2*, and the Nrf2 pathway genes (*KEAP1*,* NHE2L2*, and* CUL3*) were identified as pRCC driver mutations, often found in tumour subclones. ARID2 forms the subunit of the SWI/SNF chromatin-remodeling complex and is functionally related to ARID1A, while the NrF2 pathway protects cells against oxidative stress and regulates cell survival [161, 162]. However, less than 10% of pRCC harboured these mutations [161]. Most genetic changes were in the form of somatic copy number alterations, which were predominantly copy number gains, but the genes associated with these copy number alterations have yet to be identified [161].

Other chromosomal aberrations in pRCC are as listed in Table 1. Among these, loss of 9p may be a promising prognostic marker as Gunawan et al. and Klatte et al. associated the loss with higher stage, lymph node involvement, and increased risk of death from pRCC [137, 138]. Allelic alteration of 3p, which is characteristic of ccRCC, is also found in pRCC, although the incidence is lower [137, 185]. Loss of 3p in pRCC was associated with worse prognosis such as higher T stage and grade, lymph node involvement, distant metastasis, larger tumour size, and worse survival [137]. 

*Differential Diagnosis*

*(i) Type 1 and 2 pRCC*. Besides histological differences, types 1 and 2 pRCC may have some distinguishable patterns of chromosome aberrations. Some studies showed no difference in polysomies 7 and 17 frequency in types 1 and 2 tumours [132, 135] but others reported higher frequency of chromosomes 7 and 17 gains in type 1 compared to type 2 [137, 138, 158, 184–186]. Additionally, type 2 tumours have been shown to contain more chromosomal irregularities than type 1 tumours [137, 138, 186]. For example, type 2 tumours have a higher frequency of chromosomes 3p, 8, and 18 losses and 1q, 2, and 8q gains [137, 138, 184, 186]. However, no specific chromosome marker that can distinguish the two pRCC types has been identified. 

*(ii) Metanephric Adenoma*. Metanephric adenoma is a rare neoplasm of the kidney which has morphological similarities to pRCC. The tumours comprise of small basophilic cells arranged in a tubulopapillary pattern [207]. Most metanephric adenomas are benign with good prognosis but metastases have been reported in a few cases [208, 209]. Reports of chromosomes 7 and 17 gains and chromosome Y loss are conflicting in metanephric adenoma. Brown et al. found chromosomes 7 and 17 gains and sex chromosome loss in most of the 11 metanephric adenoma tumours analysed [210]. However, other more recent studies have reported no abnormalities in chromosomes 7, 17, X, or Y [207, 211, 212]. Brunelli et al. suggested that Brown et al. may have chosen a low threshold for chromosomal gains without normal tissue controls, resulting in the overestimation of chromosome gains [212]. Other genetic aberrations in metanephric adenoma are gains of chromosome 19 and deletions in chromosome 2 [211, 213–215].

Recently, an activating missense mutation in the* BRAF* gene was discovered, which could be specific to metanephric adenoma [215, 216]. The substitution of valine for glutamic acid in the* BRAF* gene results in the V600E variant protein, which has increased activation potential compared to the wild type BRAF [216]. It is interesting to note that the* BRAF* gene is located at 7q34, but no other RCC subtypes show* BRAF* mutation, except for one detection in a pRCC [216–218]. The BRAF protein regulates the MAPK/ERK kinase pathway, promoting cell proliferation and cell survival [219]. Due to the benign nature of metanephric adenoma, the constitutive activation of the MAPK/ERK kinase pathway may be inhibited by p16, which is overexpressed in metanephric adenomas [216]. The p16 tumour suppressor protein was suggested to be activated as a self-limiting mechanism to the MAPK/ERK kinase pathway [216].

*(iii) Clear Cell Tubulopapillary RCC*. As mentioned previously, clear cell tubulopapillary RCC lacks gains in chromosomes 7 and 17 and loss of chromosome Y frequently found in pRCC [126]. 

*(iv) Mucinous Tubular and Spindle Cell Carcinoma (MTSCC)*. Mucinous tubular and spindle cell carcinoma (MTSCC) is a newly recognized RCC subtype in the World Health Organization (WHO) 2004 classification [220]. The prognosis for MTSC is generally favourable as tumours are usually of low pathological stage at diagnosis [221]. It shares some immunohistochemical and histological features with pRCC, particularly the type 1 pRCC variants [221]. FISH analysis found no gains of chromosomes 7 and 17 and loss or chromosome Y in ten MTSCC tumours [222]. However, separate analyses by other groups showed gains in chromosomes 7 and/or 17 in their MTSCC cases [223–226]. Hence, chromosomes 7 and 17 status may not be suitable for differentiating pRCC and MTSCC. The loss of chromosome Y has only been reported in one MTSCC [226]. Other genetic alterations reported in MTSCC include losses of chromosomes 1, 4, 6, 8, 9, 13, 14, 15, and 22 [227].

*(v) Xp11.2 Translocation RCC*. This subtype, like MTSCC, was a new addition in the WHO 2004 classification [220]. Xp11.2 translocation RCCs predominantly affect children and adolescents but are found in adults as well [228]. Clinically aggressive metastatic cases have been reported [228, 229]. Xp11.2 translocation RCC cells have mixed papillary or nested structure and eosinophilic cytoplasm which may be mistaken for pRCC. Xp11.2 translocation RCCs are characterised and identified by balanced translocations of the transcription factor E3 (*TFE3*) gene on chromosome Xp11.2, resulting in gene fusions of the* TFE3* gene [230, 231]. Depending on the breakpoint of the reciprocal gene, there are six known different* TFE3* gene fusion combinations [230]. The gene fusions lead to overexpression of TFE3 protein and immunoreactivity with TFE3 protein is a distinguishing feature of Xp11.2 translocation RCCs [232].

## 4. Chromophobe Renal Cell Carcinoma (chRCC)

Chromophobe RCC, arising from the intercalated cells of the renal collecting ducts, represents 5% of RCC cases. Histologically, chRCC can be grouped into the classic, eosinophilic, or mixed variants. Chromophobe RCC cells are large and polygonal with distinct cell borders and irregular, wrinkled nuclei. The classic variant has pale, finely granular cytoplasm while the eosinophilic variant has granular eosinophilic cytoplasm [201]. Clinical outcome is similar for the different variants [233]. Compared to other RCC subtypes, chRCC has a more positive clinical outcome as it is less likely to metastasize [233, 234]. Metastasis at presentation is approximately 2.5–2.8% and disease progression after surgical resection occurs in 4.1–16.3% of chRCC patients [233, 235].

### 4.1. Birt-Hogg-Dubé (BHD) Gene

Hereditary chRCC is found in individuals with Birt-Hogg-Dubé syndrome (BHD). BHD is an autosomal dominant disorder characterised by benign skin lesions (fibrofolliculomas, trichodiscomas, and acrochordons), renal tumours, pulmonary cysts, and spontaneous pneumothorax. Renal tumours of different histologies such as ccRCC, pRCC, chRCC, and oncocytoma have been reported in BHD sufferers with chRCC and oncocytomas being the predominant types [204]. Germline mutation of the BHD or folliculin (FLCN) gene was discovered and mapped to chromosome 17p11.2 in families with BHD syndrome [205, 236]. LOH, frameshift, or missense mutations inactivate the* BHD* gene, decreasing BHD mRNA levels and folliculin protein expression [237, 238]. The function of the folliculin protein has not been completely elucidated but studies have shown that folliculin plays a role in mTOR complex 1 (TORC1) regulation [239, 240]. Kidney specific BHD knockout mice developed polycystic kidneys with upregulation of the Akt-mTOR signalling pathway, providing a link between BHD loss and renal oncogenesis [239, 240]. Unlike the* VHL* gene in ccRCC,* BHD* mutation is rarely reported in sporadic renal tumours; hence the role of folliculin in sporadic RCC is unclear [241, 242]. However, Gad et al. reported* BHD* mutations in 10.9% of chRCC and 5.6% of oncocytomas from their case series of sporadic renal tumours [191].

### 4.2. Other Genetic Aberrations

Common genetic alterations found in sporadic chRCC are the LOH at chromosomes 1, 2, 6, 10, 13, 17, and 21 [187, 188, 243, 244]. There is no difference in chromosomal loss pattern between eosinophilic and classic variants of chRCC [189]. One frequently mutated candidate gene identified in sporadic chRCC is* TP53* at 17p13.1 [191–193]. The* TP53* TSG, which is commonly implicated in cancers, regulates cell cycle arrest, apoptosis, and cell differentiation, preventing impaired DNA from being passed on to the daughter cells [245]. Davis et al. identified mutations of PTEN in chRCC but an earlier study by Sükösd et al. found no* PTEN* mutation in chRCC [192, 246]. Currently, no other candidate genes have been confirmed yet. Information on the prognostic value of genetic alterations in chRCC is scarce, possibly because of the low metastasis rate and good prognosis in chRCC. Gains of chromosomes 1–4, 6–12, 14, 15, and 17 were associated with sarcomatoid transformation in chRCC but there was no relation between chromosome change and the Paner grading system [61, 190]. This grading system, proposed by Paner et al., provides superior prognostic value in chRCC, compared to the Fuhrman grading system commonly used for ccRCC and pRCC [247].

*Differential Diagnosis*

*Renal Oncocytoma*. Chromophobe RCC and RO pose a diagnostic challenge as both tumours have morphological overlaps. Correct diagnosis is important because RO is largely benign while chRCC is malignant. Losses of chromosomes 2, 6, 10, 13, 17, and 21, found in up to 93% of chRCCs, are not features of ROs and could be used to differentiate the two tumour types [243, 244, 248].

## 5. Renal Oncocytoma (RO)

Renal oncocytomas are benign neoplasms accounting for 3–7% of renal neoplasms. Originating from the collecting ducts, RO cells are arranged in a nested, tubular, or trabecular architecture with abundance of granular eosinophilic cytoplasm and round uniform nuclei [4]. To date, there has only been one histologically confirmed case of metastatic RO [249].

### 5.1. Genetic Aberrations

Similar to chRCC, BHD syndrome predisposes affected individuals to develop RO, but BHD mutation is seldom found in sporadic cases [241]. Other genetic alterations characteristic of ROs are losses at chromosomes 1, 14, and Y, as well as chromosome rearrangement at 11q13 [194, 196, 197, 244, 250]. Rearrangement of cyclin D1 (*CCND1*) gene has been linked to the translocation at 11q13 [198, 250]. ROs with rearrangement at 11q13 have been reported along with overexpression of cyclin D1, the protein product of* CCDN1* gene [198, 250]. Cyclin D1 is involved in the regulation of cell cycle in the G1-S phase and is found to be overexpressed in a few tumours such as B-cell lymphoma, breast cancer, and squamous cell carcinoma [251]. Dysregulation of cyclin D1 may contribute to the overproliferation of cells leading to RO formation. 

*Differential Diagnosis*

*Chromophobe RCC.* As illustrated previously, losses of chromosomes 2, 6, 10, 13, 17, and 21 in chRCC distinguishe RO from chRCC. Both RO and chRCC contain loss of chromosome 1. It was proposed that chromosome 1 loss may represent a common early event in the tumourigenesis of both RO and chRCC [197, 248]. Additional losses of chromosomes 2, 6, 10, 13, 17, and 21 in RO may lead to malignant transformation to chRCC [197, 248, 252].

## 6. Collecting Duct Renal Cell Carcinoma (CDRCC)

Collecting duct RCC is an uncommon histological subtype, accounting for 0.4–1.8% of all RCCs [253]. CDRCC cells have a tubulopapillary or hobnail structure, with eosinophilic cytoplasm and large nuclei. CDRCC is clinically aggressive with a higher metastatic rate and poorer prognosis compared to other RCC subtypes [253]. Patients are often diagnosed at an advanced stage, including 32–45% with distant metastasis and 42–44% with positive lymph nodes [253, 254].

### 6.1. Genetic Aberrations

A cytogenetics study found monosomies 1, 6, 14, 15, and 22 in three CDRCC tumours [202]. Another recent study in 29 CDRCC tumours reported genetic losses at 8p, 16p, 1p, and 9p and gains in 13q [201]. Loss of chromosome 3 is rarely reported in CDRCC whereas frequent loss of chromosome 1 is similarly seen in RO and chRCC, both originating from the renal collecting duct [255]. Hence, loss of chromosome 1 might be more characteristic of renal tumours from the collecting duct whereas chromosome 3p loss is more typical of renal tumours from the proximal tubule [256]. Steiner et al. detected a region of genetic loss at 1q32.1-32.2 in 69% of CDRCC but no TSG has been identified yet [256].

Due to the rarity of CDRCC, knowledge on genetic aberrations and the role they play in pathogenesis of the tumour is lacking. A better understanding of the contribution of genetic alterations in CDRCC would be interesting because of the aggressive behaviour of the disease.

*Differential Diagnosis*

*(i) Upper Urinary Tract Urothelial Carcinoma*. Upper urinary tract urothelial carcinoma (UUTUC) and CDRCC display some similarities in histology and immunoreactivity and hence may present some difficulties in diagnosis [257]. A cytogenetic comparison of UUTUC and CDRCC showed distinct genetic alterations in the two tumour types [201]. CDC showed frequent losses at 8p, 16p, 1p, and 9p and gains at 13q while UUTUC showed losses at 9q, 13q, and 8q and gains at 8p [201]. 

*(ii) Renal Medullary Carcinoma*. Renal medullary carcinoma (RMC) is a rare, highly aggressive form of kidney cancer with predominance in individuals with sickle cell trait. Renal medullary carcinoma and CDRCC are both aggressive with some similarities in morphology and immunoreactivity [258]. Swartz et al. analysed 9 RMC tumours for genetic aberrations but found only loss of chromosome 22 in one case [259]. The lack of genetic losses or gains was surprising given that RMCs are aggressive and are closely associated with sickle cell trait [259]. Gatalica et al. evaluated 3 patients with RMC for chromosomal abnormalities and compared their results with findings of other publications on RMC and CDRCC cytogenetics [260]. They concluded that no consistent chromosomal abnormalities were observed in RMCs or CDRCC [260]. However, limited studies due to the rarity of both tumour types may have contributed to these inconsistent findings. 

*(iii) Tubulocystic Renal Cell Carcinoma*. Tubulocystic RCCs are characterised by multiple tubules and cysts with bubble-wrap appearance, eosinophilic cytoplasm, and presence of hobnail cells [261]. It was originally thought to originate from the collecting duct and was classified as low grade CDRCC due to its indolent clinical behaviour [262]. Recent analyses have suggested that tubulocystic RCC might be of proximal tubule origin from its immunostaining profile and ultrastructural features (Pax 2 positivity and presence of short microvilli with brush border structure) [261, 263]. Zhou et al. and Al-Hussain et al. reported either gains of chromosomes 7 or 17 and loss of chromosome Y in their case series of tubulocystic RCC tumours, indicating similarities to pRCC [263, 264]. The tumours that they analysed consisted of areas with coexisting pRCC or CDRCC morphologies [263, 264]. However, another case series by Amin et al. found none of these chromosomal anomalies typical of pRCC [261]. A possible reason for this discrepancy could be that the tumours analysed by Amin et al. were purely tubulocystic RCC without coexisting pRCC morphology. More studies are required for a clearer understanding of genetic changes in these tumours.

## 7. Conclusions

Each RCC subtype has a distinctive pattern of genetic aberrations, although there are some overlaps in chromosomal and genetic changes. These genetic changes may play an important role in tumourigenesis and affect the progression or prognosis of the tumour. However, most genetic studies were concentrated on ccRCC and pRCC as they are two most common forms of RCC. More studies on other RCC subtypes are needed to identify the specific genetic changes which may be involved in tumourigenesis. Recent genetic studies have employed gene sequencing or gene expression profiling for discovery of novel gene mutations which could identify possible differentially expressed proteins in RCC subtypes. These proteins can be part of an immunoreactivity panel for diagnosis of RCC subtypes. Hence, detection of genetic or chromosomal changes could be a useful diagnostic or prognostic tool as adjunct to conventional immunohistochemistry and histology. Identification of frequently mutated genes and affected signalling pathways also allows for development of new therapeutic targets or personalised targeted therapy for better management of advanced RCC.

## Figures and Tables

**(a):** 

Chromosome	Type of genetic alteration	Candidate gene	Incidence	Function of protein	Prognosis
3p25-26	LOH, hypermethylation, mutations	*VHL *	Found in 57–91% of tumours [30, 31, 37, 40, 163–165]	pVHL targets HIFa degradation; nonfunctioning pVHL does not degrade HIFa leading to angiogenesis	No clear association between *VHL* status and tumour grade and stage or survival of patients [31, 36–38, 40, 165]

3p14.2	LOH, translocation, hypermethylation	*FHIT* contains chromosome fragile site *FRA3B *	Aberrations in 69–90% of ccRCC [62, 64, 78, 166]; 51–90% of ccRCCs showed reduced or absent FHIT protein expression [72, 74, 77]	FHIT protein is involved in apoptosis and proliferation [167–170]	*FHIT* loss, an early event in RCC; correlation of inactivation with lower grade and stage as well as better survival [73, 74, 77, 78]

3p21.3	Hypermethylation	*RASSF1A *	Methylation in 23–91% ccRCC tumours [88, 171]	RASSF1A promotes cell cycle arrest, apoptosis, and microtubule stability [79]	Hypermethylation of the *RASSF1A* promoter is significantly associated with advanced stage, grade, and worse cancer specific survival [85, 86]; loss of RASS1A protein is found in most ccRCC; but tumours with RASSF1A immunopositivity associated with higher stage, grade, and worse survival [87]

3p21	Truncating and missense mutations	*PBRM1 *	Mutations in 29–41% of ccRCC tumours [90, 92, 105, 109]	BAF180 subunit of the SWI/SNF (switch and sucrose nonfermentable) chromatin remodeling complex; SWI/SNF complex regulates cell differentiation, proliferation, replication, transcriptional regulation, and DNA repair [90, 172, 173]	Due to contradictory findings, relationship of *PBRM1* status and prognosis is still unclear [103–106]
1p36.11	Copy number loss	*ARID1A *	Copy number loss in 16% of patients with ccRCC [93]	BAF250a subunit of SWI/SNF complex	Low ARID1A mRNA and BAF250a immunostaining associated with higher stage, grade, and worse disease-free and disease specific survival [93, 111]
3p21.3	Truncating and missense mutations	*BAP1 *	Inactivated in 6–15% of ccRCC [91, 92, 105, 109]	BAP1 is involved in cell cycle regulation [174]	*BAP1* is an indicator of worse prognosis [91, 105, 107–110]
3p21.31	Truncating and missense mutations	*SETD2 *	Mutation in 8–16% of tumours [92, 105, 109]	SETD2 is a histone methyltransferase controlling transcriptional regulation [175]	*SETD2* mutation associated with worse disease specific survival [105]
Xp11.2	Truncating and missense mutations	*KDM5C *	Mutation in 4–8% of tumours [92, 109]	Histone demethylase, transcriptional regulation [176]	Tumours with mutations in *BAP1*, *SETD2*, or *KDM5C* are significantly associated with higher stage [92]

5q21.2~q21.3	Copy number loss or gain	NA	Copy number gain in 32–34% and loss in 52–56.2% of tumours [112, 113]	NA	Significant association of loss at 5q21.2~q21.3 with high grade tumours in patients with 3p loss [113]
5q22~q23	Copy number loss or gain	NA	Gains in 48–52.4% and loss in 42.9–46% of tumours [112, 113]	NA	Gain at 5q22.3~q23.2 associated with smaller, low grade tumours and better disease specific survival; loss at 5q22.3~q23.2 significantly related to larger, high grade tumours and poor disease specific survival [112, 113]
5q31–qter	Copy number gain	NA	Gain in of 56.8% ccRCCs [112]	NA	Gain of 5q31–qter had better overall survival compared with patients without gain of 5q [114]

8p	LOH	NA	LOH in 32-33% of RCC tissue specimen [115, 116, 177]	NA	LOH on chromosomes 8p and 9p, a significant predictor of recurrence [115]; LOH of 8p correlated with higher stage and grade [116]

9p	LOH	Possible candidate gene at 9p21 *CDK2NA/ARF* [118, 119]	LOH in 14–33% of RCC tissue specimen [115, 117, 119]	p16 is the protein product of *CDK2NA* which regulates cell cycle [118, 119]	Associated with high grade and stage, lymph node involvement, metastases, recurrence, and worse survival [115, 117–120]

14q	LOH	Possible *HIF1A* at 14q23.2 [121]	Loss in 38–55% of tumours [116, 121, 178, 179]	HIF1a is a transcription factor which regulates cellular response during hypoxia, for example, angiogenesis	Correlation of LOH at 14q with advanced stage, grade, larger tumour size, recurrence, and shorter cancer specific survival [116, 121, 178–180]

Chromosome 7	Polysomy	NA	Polysomy 7 in 9.5–56.2% ccRCC [133, 181]	NA	Polysomy 7 associated with higher tumour grade, stage, and higher proliferative rate [133]

NA indicates not available.

**(b):** 

Chromosome	Type of genetic alteration	Candidate gene	Incidence	Function of protein	Prognosis
Chromosome 7	Polysomy	NA	Polysomy 7 in 50–100% pRCC [133, 181, 182]	NA	Polysomy 7 is not correlated with survival, clinical features, or proliferation rate of pRCC [132, 133, 136, 137]

7q31	Missense mutation, gene duplication	*MET *	*MET* mutation present in 5–21.6% of sporadic pRCC [8, 108, 139, 141]; copy number gains in 46% of type II pRCC and in 81% of type I pRCC [108]; strong c-met protein expression in 80–90% of sporadic pRCC [143, 144]	The c-met protein is involved in cell proliferation, motility, differentiation, invasion, and angiogenesis [142, 143]	One study showed association of increasing tumour stage with c-met expression and a trend of better overall survival in patients with no c-met expression in tumours [143]

Chromosome 17	Polysomy	NA	Gains in 14.3–95.5% of pRCC [132, 134, 137, 158, 183]	NA	Trisomy 17 associated with better prognosis (lower stage, less nodal involvement and metastases, longer survival) [137, 138]; Balint et al. showed no link with tumour size and grade [132]

Chromosome Y	Loss	NA	Affects 71–87% of tumours in men [137, 138, 158, 184]	NA	Loss of chromosome Y not linked to pathological variables and survival [137]

Chromosome X	Loss	NA	Loss involving Xp in 28% and Xq in 36% of pRCC tumours [158]	NA	Losses of chromosome Xp associated with short patient survival [158]

3p	loss	NA	Allelic changes in 14–37.5% of tumours [137, 185]	NA	Loss of 3p associated with higher stage and grade, lymph node involvement, distant metastasis, larger tumour size, and worse survival [137]

Gains of 1q, chromosomes 12, 16, and 20; losses of 1p, 4q, 5q, 6q, 8p, 9p, 11, 13q, 14q, and 18 [135, 137, 138, 158, 186]					Loss of 9p associated with higher stage, larger tumour size, metastasis, lymph node involvement, recurrence, and decreased survival [137, 138]; losses of 8p and chromosome 18 correlated with higher stage, metastasis, and worse recurrence free survival [138]

NA indicates not available.

**(c):** 

Tumour type	Chromosome	Type of genetic alteration	Candidate gene	Incidence	Function of protein	Prognosis
chRCC	Losses at chromosomes 1 (73–90%), 2 (70–90%), 6 (62–96%), 10 (70–91%), 13 (51–86%), 17 (65–90%), and 21 (32–90%) [187–190]; *TP53* mutation in sporadic chRCC (24–32%) located at 17p13.1 [191–193]					No relation between chromosomal numerical changes and the Paner grading system [190]

RO	Loss or alterations of 1p (31–86%) [188, 194, 195], chromosomes Y (100% in males) [196] and 14 (15–50%) [188, 197]					NA
	11q13	Translocation	*CCND1 *	*CCND1* translocation in 11–36% of ROs [198–200]	Cyclin D1 is involved in the regulation of cell cycle, G1-S phase, and cell proliferation	NA

CDRCC	Reported losses of chromosomes 1, 1p, 6, 8p, 9p, 14, 15, 16p, and 22 and gains in 13q in CDRCC tumours [201, 202]					NA

NA indicates not available.

**Table 2:** Hereditary RCCs and their related genetic aberrations.

Tumour type	Syndrome	Clinical manifestation	Chromosome	Candidate gene
ccRCC	Von Hippel-Lindau (VHL)	Retinal, cerebellar and spinal hemangioblastoma; pheochromocytoma; pancreatic, epididymal and renal cysts; hemangiomas of the adrenals, liver, and lungs; endolymphatic sac tumours; cystadenoma of the epididymis or broad ligament and ccRCC (40–75%) [19, 20]	3p25	*VHL *
	Constitutional chromosome 3 translocation	ccRCC	Various breakpoints in chromosome 3	Possible breakpoint associated genes: *NORE1*, *LSAMP*, *DIRC1*, *DIRC2*, *DIRC3*, *FHIT*, *TRC8* Possible known common gene lost from translocation: *VHL *

pRCC	Hereditary papillary RCC (HPRC)	pRCC	7q31	*MET *
	Hereditary leiomyomatosis and RCC (HLRCC)	Cutaneous leiomyomas, uterine fibroids, type II Renal tumours (14–18%) for example pRCC and CDRCC [149, 152, 154, 203]	1q42-43	*FH *

chRCC or RO	Birt-Hogg-Dubé (BHD)	Skin fibrofolliculomas, pulmonary cysts, spontaneous pneumothorax, and renal cancer (20–29%) (chRCC, RO, ccRCC and pRCC) [204–206]	17p11.2	*BHD*, *FLCN *
